# Percutaneous Mechanical Thrombectomy Using Rotarex® S Device in Acute Limb Ischemia in Infrainguinal Occlusions

**DOI:** 10.1155/2017/2362769

**Published:** 2017-05-07

**Authors:** Samuel Heller, Jean-Claude Lubanda, Petr Varejka, Miroslav Chochola, Pavel Prochazka, David Rucka, Sylvie Kuchynkova, Johana Horakova, Ales Linhart

**Affiliations:** 2nd Department of Medicine, Department of Cardiovascular Medicine, First Faculty of Medicine, Charles University and General University Hospital in Prague, Prague, Czech Republic

## Abstract

**Purpose:**

To evaluate the effectiveness of percutaneous mechanical thrombectomy using Rotarex S in the treatment of acute limb ischemia (ALI) in infrainguinal occlusions in a retrospective study of patients treated in our institution.

**Methods:**

In this study, we identified a total of 147 ALI patients that underwent mechanical thrombectomy using Rotarex S at our institution. In 82% of the cases, percutaneous thrombectomy was used as first-line treatment, and for the remainder of the cases, it was used as bailout after ineffective aspiration or thrombolysis. Additional fibrinolysis and adjunctive aspirational thrombectomy were utilized for outflow occlusion when required. Procedural outcomes, amputation rate, and mortality at 30 days were evaluated.

**Results:**

Of the 147 patients treated with mechanical thrombectomy, Rotarex S was used as first-line treatment in 120 cases and as second-line treatment in 27 cases. Overall, we achieved 90.5% procedural revascularization success rate when combining mechanical thrombectomy with limited thrombolysis for severe outflow obstruction, and 1 death and 3 amputations were observed. We achieved primary success in 68.7% of the patients with the mechanical thrombectomy only, and in 21.8% of the patients, we successfully used additional limited thrombolysis in the outflow. The overall mortality was 0.7% and amputation rate was 2% at 30 days.

**Conclusion:**

Percutaneous mechanical thrombectomy as first-line mini-invasive treatment in infrainguinal ALI is safe, quick, and effective, and the performance outcomes can be superior to that of traditional surgical embolectomy.

## 1. Introduction

Acute limb ischemia (ALI) is a potentially life-threatening condition. For decades, surgical treatment was the standard therapy for ALI [1, 2]. Catheter-directed intra-arterial thrombolysis (CDT) was later introduced as an alternative mini-invasive treatment modality [3, 4]. Percutaneous aspiration thrombectomy (PAT) using simple catheters with 60 cc syringe was often combined with CDT to remove older thrombus resistant to thrombolytic therapy [5, 6]. Blindly performed surgical thrombectomy from the common femoral artery is not always effective in cases of infrainguinal occlusion often involving popliteal trifurcation, and selective embolectomy from pedal arteries is usually not feasible [7]. Thus, a means for minimally invasive and quick removal of a relatively large thrombus mass appears as a necessity, especially in the aging population. This approach is feasible by using percutaneous mechanical thrombectomy devices [8, 9]. They enable rapid removal of thrombus that is comparable to surgical embolectomy with the advantage of selective angiography controlled procedure, mostly followed by other adjunctive endovascular procedures such as balloon angioplasty, stenting, selective PAT from tibial arteries, and if necessary adjunctive local intra-arterial fibrinolysis [10]. These procedures require only local anesthesia.

When surgical approach is used as first-line treatment, and if it is ineffective for the distal tibial and pedal arteries, the possibility of adjunctive thrombolysis for outflow obstruction is limited because of high risk of bleeding from surgical wounds.

To keep all possibilities open and be able to remove large amount of thrombus at the same time, the percutaneous mechanical thrombectomy first approach seems to be an attractive option.

Among other thrombectomy devices on the market, Rotarex S (Straub Medical AG) was used in our institution since 2008 due to its ability to aspirate both fresh and old thrombus [11]. In this study, we aimed to evaluate the effectiveness of percutaneous mechanical thrombectomy using Rotarex S in the treatment of ALI in infrainguinal occlusions in a retrospective study of patients treated in our institution.

## 2. Material and Methods

### 2.1. The Study Cohort

We present retrospective data of patients with ALI who were referred to our tertiary university center from several surrounding hospitals in our area. All patients underwent digital subtraction angiography (DSA) as the definitive diagnostic procedure. Among the patients treated in our institution for ALI from 2008 to 2015, we were able to identify 147 consecutive patients that were treated by percutaneous mechanical thrombectomy. This study presents the data of this study population (Table 1). Procedural outcomes (success and failure), amputation rate, and mortality at 30 days are reported.

### 2.2. Procedure Description

All patients were evaluated by endovascular specialist and vascular surgeon immediately after initial digital subtraction angiography (DSA) and the mode of therapy was chosen consensually. The puncture site and approach were chosen on the base of clinical examination and orientation duplex ultrasound. In some patients, who were sent from referring hospitals after previous diagnostic workup, the access site was chosen on the basis of CT angiography or MR angiography. When possible, the ipsilateral approach was always preferred because it makes all additional procedures such as percutaneous aspirational thrombectomy (PAT) and PTA of distal tibial arteries much easier. All the procedures were performed by five experienced endovascular specialists from our center.

We considered primary success to be a complete recanalization of the occluded segment with satisfactory outflow and good capillary filling of the distal parts of the foot without any major or obstructing residual thrombi either in the treated segment or in the outflow tract.

### 2.3. Mechanical Thrombectomy Using Rotarex S

The Rotarex S aspiration thrombectomy catheter works over the 0.018-inch wire. The tip of the catheter connected to the Archimedes screw rotates and fragments the thrombus; the fragments are then sucked into the catheter from the opening at the tip and transported out of the body with blood into a collecting bag, where the aspirated fluid can be checked and measured. The whole system consists of three parts: a driving unit connected to the motor unit inserted in a sterile plastic bag, which is magnetically connected to a single-use disposable catheter.

Since the Archimedes screw and the tip of the catheter rotate at a speed of 40,000 revolutions per minute to create sufficient vacuum to aspirate the thrombus, it needs to be cooled by blood. Depending on the size of the system, Rotarex S can aspirate up to 1.5 mL/sec. The whole system can be set up within two minutes.

**Figure 1:**
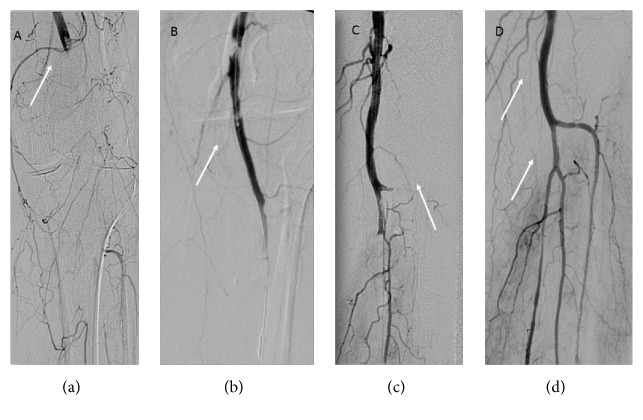
Acute occlusion of popliteal and tibial arteries treated by mechanical thrombectomy with Rotarex S, PAT, and CDT. Panel (a): white arrow points to total occlusion of popliteal and tibial arteries. Panel (b): partial recanalization of popliteal artery after first crossing with Rotarex S. Panel (c): recanalization of popliteal artery after third crossing with Rotarex S. Panel (d): final result of recanalization after adjunctive CDT and PAT. White arrows showing all patent arteries below the knee.

The occlusion is crossed by a wire and a diagnostic catheter. Then angiography is then performed through the tip of the catheter to ensure that it is safe within the lumen and beyond the occlusion; then through the catheter, a special 0.018-inch 260 cm long wire is inserted. Over this wire, the flushed Rotarex S system is introduced just above the occlusion. A slow aspiration is performed, and depending on the size of the vessel and the thrombus load, the 6F or 8F system is used. One or two passes of the device through the occlusion are usually enough to reestablish good flow in the vessel (Figure 1).

During the procedure, anticoagulation is recommended as in any other angioplasty procedure, and we prefer to saturate patients with antiplatelet therapy during or just after the procedure.

### 2.4. Additional Fibrinolysis

Additional fibrinolysis was administered at the decision of the operator. The patient was placed at a dedicated angiology intensive care unit with experienced personnel, where the puncture site, laboratory parameters, and clinical status of the patient and the limb were closely observed and any other arising medical conditions, such as pain, electrolyte, and acid base imbalance, reperfusion syndrome, and renal impairment, were treated appropriately. Fibrinolysis was administered through a multihole catheter inserted into the residual thrombus following our institution's standard intra-arterial fibrinolytic protocol (1 mg of rtPA-Actilyse™ per hour, usually 20 mg of rtPA diluted in 500 ml of saline administered by infusion pump). Simultaneously, unfractionated heparin was administered to keep aPTT between 60 and 90 seconds. The coagulation parameters INR, aPTT, and fibrinogen levels were monitored every 6 hours and a blood count was done every 12 hours. There were two modes of rtPA administration: either it was administered only into a multihole catheter placed in the popliteal artery or the dose was divided into a kink resistant sheath and into a distally placed catheter, so that the thrombolytic solution would be infused at two points into the arterial system, depending on the localization of residual thrombi. The angiographic effect was usually checked within 10–20 hours.

### 2.5. Other Adjunctive Endovascular Procedures

Adjunctive aspirational thrombectomy was usually performed through 45 cm long Destination Guiding Sheath (Terumo Corp.) size 6F to 8F, with a removable valve placed ipsilaterally into the popliteal artery. Radiofocus Guidewire M (Terumo Corp.) or V-18 Control Wire (Boston Scientific Corp.) was used to engage the occluded tibial arteries and then a simple aspiration catheter or multipurpose guiding catheter diameter depending on the size of the vessel with 60 ml syringe was used to aspirate the residual thrombus. Stenting and PTA were performed in the same way as in routine standard procedures. To avoid displacement or damage by aspiration catheters, stent placement was usually the last step of the procedure.

At the end of procedure, a closure device was used and the patient was subsequently treated with low molecular weight heparin and antithrombotic therapy—aspirin or clopidogrel. If the cause of ALI was embolism, then the patient was set on lifelong anticoagulation therapy.

## 3. Statistical Methods

Continuous variables are presented using median or average, as appropriate based on their distributions. Categorical variables are summarized using proportions. Only methods of descriptive statistics are provided, and no hypothesis testing was needed. Statistica version 12 (StatSoft, Prague, CZ) was used in the analysis.

## 4. Results

We treated a total of 147 ALI patients with mechanical thrombectomy and median age of 69 years (26 to 96 years of age), 93 men and 54 women mostly (95%) for infrainguinal occlusions. The median time of the interventional procedure was 1 hour and 10 minutes. We used Rotarex S as first-line treatment in 120 (84%) cases. In 27 (18%) cases, we used mechanical thrombectomy as rescue therapy after ineffective CDT, where simple catheter aspirational thrombectomy was no longer effective, for example, in cases where the occluded segment was too long and the artery was relatively large. We also successfully treated 7 suprainguinal occlusions in cases where surgical therapy could not be performed for various reasons (Table 1).

The clinical stages of acute limb ischemia are detailed in Table 2, and the causes of occlusion are documented in Table 3.

Overall 46 (31%) of the patients were considered to have severe limb ischemia as they presented with severe motor deficits (27% in class IIb and 4% in class IIb-III). We achieved primary success in 68.7% of the patients with mechanical thrombectomy and additive simple aspirational thrombectomy from tibial arteries. In 21.8% of the patients, we used additional thrombolysis in outflow obstruction for diffused or distal residual thrombi in tibial or pedal arteries.

Out of the 13 unsuccessfully treated patients of our cohort, 7 had successful rescue surgery and 3 required amputation and two could be managed conservatively. One patient, an 82-year-old lady with SVS class IIb acute limb ischemia with low cardiac output and renal failure, died of multiorgan dysfunction.

Although we have encountered some complications throughout the procedures (Table 4), overall, with percutaneous mechanical thrombectomy and additional thrombolysis combined, we achieved 90.5% procedural revascularization success rate, 2% amputation rate, and 0.7% mortality rate (Table 5).

## 5. Discussion

Introduction of percutaneous mechanical thrombectomy is a logical step to further improve ALI treatments in the field of evolving endovascular therapy [12, 13]. Up until now this approach has not been clearly supported in the guidelines as first-line therapy. Our data show that we are able to save more limbs at risk with less complications in comparison to the results of well-established Rochester, STILE, and TOPAS trials comparing catheter-directed thrombolysis to standard surgical therapy in acute limb ischemia (Table 6) [14–16].

Through this comparison, we can see that the approach of percutaneous mechanical thrombectomy as a primary choice combined with overnight fibrinolysis (21.8% of our cases) led to 90.5% revascularization success rate whereas the aspiration technique only, in our setting, led to almost 70% success rate. The primary success rate could be higher if more attempts for tibial artery aspiration (PAT) were performed, but in our institution CDT is used routinely at our dedicated angiology intensive care unit and our team is familiar with local thrombolysis overnight. Therefore, local thrombolysis for distal outflow obstruction was usually preferred over excessive aspiration. There are also some advantages of thrombolytic administration if the clinical status of the patient allows for this approach. It saves time at the angiographic table, creates less dissection in often sclerotic tibial arteries or in young patients with frequent severe spasms, and also has beneficial effect on microvasculature and the periphery of the foot. Also it was reported that successful treatment in acute limb ischemia depends upon recanalization of the outflow and the amount of residual thrombosis [17].

On the other hand in the setting of interventional radiology suite or lack of personnel routinely used to intra-arterial thrombolysis application and difficulty to secure bed at intensive care unit, there is more pressure to make endovascular therapy of acute limb ischemia a one-stop event.

Therefore, many endovascular specialists try to remove as much thrombi as possible by first using mechanical thrombectomy and then repeated aspiration (PAT). In case of higher recanalization success, the rate of additional fibrinolysis is then lower in comparison to our study [18–20].

Previous studies showed good results with mechanical thrombectomy, but the cohorts usually consisted of patients with both acute and subacute ischemia. This is most likely due to the fact that Rotarex S can aspirate both fresh and old thrombi. From our experience, the clinical course and outcome of ALI is quite different from subacute ones, even though the angiographic pictures and procedures can be similar to some extent. Patients with acute limb ischemia are exposed to ongoing myocytal necrosis which often leads to severe systemic electrolyte disorders and can further lead to cardiopulmonary and renal impairment including arrhythmias, decreased left ventricle contractility, and renal failure. Myoglobin levels are predictive of development of compartment syndrome and acute renal failure [21, 22]. Also the cohort with severe motor deficit (classes IIb and III) comes to hospital usually in poor condition and if older often dehydrated and sometimes found at home after hours unable to walk. On the other hand, patients with subacute limb ischemia behave more similar to CLI patients. Also, the fact that they were not brought to the hospital in the acute state, but much later, confirms that they are in more stable clinical condition with at least partially preserved collateral circulation.

### 5.1. Disadvantages of Fibrinolysis

Fibrinolysis has some substantial disadvantages; it takes more time to revascularize. In our opinion, the administration of CDT requires availability of an intensive care unit, with highly trained personnel, skilled in this very specific therapeutic modality (aged patient with intra-arterial fibrinolysis). The patients need to be constantly monitored and there should be vascular surgeon available in case of bleeding complications. Many negative factors such as old age, mental status of the patient, obesity, and the extent of atherosclerotic involvement of the vessels have impact on the outcome of this otherwise well-established method, and the outcome cannot always be influenced by the treating physician (Table 7). The risk of serious bleeding in fibrinolytic studies in ALI was 5 to 12% [15].

Percutaneous mechanical thrombectomy in patients with acute limb ischemia reduces time spent at intensive care unit, reduces risks of bleeding complication, and makes endovascular therapy of acute limb ischemia a one-stage procedure in majority of the cases, which makes this procedure even more attractive in terms of cost effectiveness. In addition, as our patients were predominantly older, the less invasive the procedure, the better the outcome.

We also consider percutaneous mechanical thrombectomy a more physiological alternative treatment for arterial occlusions. When quick mechanical debulking is first performed and the thrombus is removed, the underlying lesion is usually significantly shorter than the length of the occlusion. This can translate into better long-term patency rates and higher long-term limb salvage rate.

### 5.2. Complications of the Procedure

We encountered two types of complications: the complication arising from treating acute limb ischemia and those related to endovascular therapy and the use of device. Since we had 46 patients with severe limb ischemia (classes IIb and IIb-III), 6 patients required fasciotomy for compartment syndrome, and 3 had acute renal failure of which one died due to low cardiac output and resulting multiorgan dysfunction.

Percutaneous mechanical thrombectomy also carries risks for some potential complications. We can expect the same or higher complication rate in any other endovascular procedure, with bleeding from local entry site, especially when patients need full anticoagulation therapy immediately after the procedure. Moreover, distal embolization can be encountered, and its rate depends on the operator's experience. The reported rate of distal embolization is around 8% and we encountered lower percentage in our cohort (4,8%) [14]. In our experience, it happens usually by dislodging residual thrombus during balloon postdilatation, not during the thrombectomy procedure itself.

There is also a potential risk of vessel perforation especially in case the guidewire passes subintimally, which is less probable in the setting of fresh thrombus but can be encountered more often in chronic occlusions. The risk of perforation would be higher in smaller and calcified arteries, typically when larger 8F device is used in smaller distal popliteal artery. We had 7 perforations in our series and we were able to solve the problem by endovascular means either by prolonged balloon inflation or by short stent graft implantation into the popliteal artery in 5 cases. The device also has a strong suction effect; when operating within a stent, if no sufficient blood inflow is present, the negative pressure can cause the stent to collapse and catch the struts, especially when the integrity of a stent was already disrupted. We encountered this complication requiring surgical correction only once and we consider it to be a part of our learning experience. In our cohort, we had a relatively small proportion of in-stent occlusions, probably due to our conservative policy towards stenting infrainguinally in previous years.

The 6F and 8F Rotarex S device can both be used for thrombus aspiration in the arterial system. The 6F device is recommended for smaller vessels that are 4 to 5 mm in diameter, usually corresponding to patients with smaller arteries. The 6F device is favored in cases where acute ischemia occurred in severely calcified vessels and when there is a need for aspiration in popliteal trifurcation. It is also the device of choice when additional thrombolysis is deemed necessary, because we prefer to avoid 8F sheath for additional thrombolysis administration since it relatively obstructs the inflow and increases the risk of partial thrombosis at the site of sheath insertion.

On the other hand, the 8F Rotarex device is more powerful, and if the affected arteries are relatively large, it enables the physician to make revascularization a one-stop procedure, with femoropopliteal mechanical aspiration conducted first, followed by simple tibial aspirational thrombectomy. From our experience, the critical sites, where the suction power is of importance, are the proximal cap of the thrombus, which is always harder and more strongly attached to the vessel wall, and then the end of occlusion, where sufficient suction power prevents distal embolization.

We acknowledge that there are more percutaneous thrombectomy devices (PTD) available on the market. Each of them has certain characteristics that mark their advantages and drawbacks. Some can be used for both older and fresh thrombus, like Rotarex system, or atherectomy devices like Jetstream that can also be effectively used as debulking device for older thrombus or myointimal neoplasia in in-stent restenosis. In the USA, the only FDA approved PMD is Angiojet, which works on principle of rheolytic thrombectomy. The authors have experience with older version of the device in both arteries and veins. It works over the wire, creating vacuum by Venturi effect by high speed jets of saline inside the catheter and the vacuum subsequently produces streams within the vessel lumen disrupting the thrombus, which is then sucked into the catheter and transported out of the body into a collecting bag. This device is relatively safe and low profile—6F for the arterial system. It can be also used in the power pulse mode, when it combines pulses of fibrinolytic (e.g., tPA) into the thrombus with subsequent aspiration after approximately 20 minutes when the thrombus is partially cleft. The only drawbacks from our experience are that it is less effective on older thrombus and it can produce hemolysis and macroscopic hemoglobinuria, when working longer. However, this could be usually dealt with by sufficient hydration. The data from a multicenter registry with Angiojet rheolytic thrombectomy in limb threatening ischemia show comparable results to our cohort [23, 24].

### 5.3. Study Limitations

In our study we were not able to determine the exact severity of ALI in some cases as patients were referred to our hospital with insufficient documented evaluation from local hospitals. We classified them as IIa or I, since we admit all patients with severe limb ischemia to our intensive vascular unit due to the potential threat of reperfusion syndrome. On the other hand, this cohort reflects all consecutive patients coming to a tertiary center for ALI who were not clear candidates for surgery, for example, in cases with acute suprainguinal embolic occlusion or occlusion of the femoral bifurcation. Also, this analysis is retrospective and we have only short-term (in hospital up to one month) follow-up data for most patients.

## 6. Conclusion

Mechanical thrombectomy as first-line treatment in infrainguinal ALI is safe and effective, and the performance outcomes can be superior to that of traditional surgical embolectomy. This method combined with CDT is a suitable alternative to surgical embolectomy in infrainguinal territory with lower risks of amputation, lower morbidity, and mortality rates as compared to previous studies. This approach reduces the need to use thrombolysis and thus decreases the risk of serious bleeding complication. In our opinion, it should be embraced by the guidelines as a method of choice in experienced high volume centers for treatment of infrainguinal ALI without the involvement of femoral bifurcation.

## Figures and Tables

**Table 1:** Patient data of ALI cases treated with Rotarex.

Study population (men, women)	147 subjects (93 men, 54 women)	
Age (median)	69 years (29–96 years)	
All ALI^1^ cases treated with Rotarex	147 cases	100%
Used as first-line treatment	120 cases	82%
Used as bailout after ineffective aspiration or thrombolysis	27 cases	18%
Suprainguinal occlusions	7 cases	5%
Infrainguinal occlusions	140 cases	95%
6F device used	89 cases	60%
8F device used	58 cases	40%
Procedural time (median)	1 hour 10 minutes	
Contrast amount (average)	142 ml/procedure	
Skiascopy time (average)	20.8 minutes	

Abbreviation: ^1^ALI, acute limb ischemia.

**Table 2:** Distribution of stages of ALI in all patients treated with mechanical thrombectomy.

ALI^2^-SVS class documented	Number of patients	Percentage (%)
I	38	26%
IIa	35	24%
IIb	40	27%
IIb-III	6	4%
Undetermined (outpatients I or IIa)	28	19%

*Total*	*147*	*100%*

Abbreviations: ^2^ALI: acute limb ischemia; SVS: Society for Vascular Surgery.

**Table 3:** Causes of occlusion.

Causes of occlusion	Number of cases	Percentage (%)
Embolism	62	42%
Thrombosis	38	26%
Bypass occlusion	27	18%
Reocclusion after percutaneous intervention	13	9%
Other	7	5%

**Table 4:** Complications throughout the procedures.

Procedural complications	Number of cases	Rate
Compartment syndrome requiring fasciotomy	6	4%
Acute renal failure	3	2%
Access site hematoma	6	4%
Extravazation solved with long balloon inflation	2	1.4%
Vessel perforation solved with stent graft	5	3.4%
Vessel perforation requiring surgical therapy	1	0.7%
Distal embolization	7	4.8%
Stent capture	1	0.7%

**Table 5:** Procedural success and failure.

Procedural success	Number of cases	Percentage (%)	Total
Primary success	101	68.7%	*90.5%*
Success with additional fibrinolysis for outflow occlusion	32	21.8%	

*Nonsuccessful (total)*	13	8.8%	*8.8%*
(i) Rescue surgery	7	4.8%	
(ii) Amputation	3	2%	
(iii) Other	3	2%	

*Mortality*	1^3^	0.7%	*0.7%*

^3^82 years old, SVS class IIb with low cardiac output and renal failure.

**Table 6:** Comparison of our data to historical clinical trials.

	Mortality at 30 days	Amputation rate	Success of revascularization
Rochester trial	16%	18%	70%
STILE trial	4%	5%	82%
TOPAS trial	5%	2%	80%
Our study^4^	0.7%	2%	90.5% (68.7% + 21.8%)

^4^Mechanical thrombectomy as first-line treatment combined with overnight fibrinolysis.

**(a):** 

Comorbidities/risk factors	Number	%
Diabetes mellitus	44	30%
Congestive heart failure	14	9,50%
Atrial fibrillation	36	24,40%
Coronary artery disease documented	51	34,60%
Smoking		
Current smoker	42	28,50%
Positive history of smoking	35	23,80%
Preexisting renal insufficiency	20	13,60%
COPD	17	11,50%
Dyslipidemia	63	42,80%
Atrial hypertension	104	70,70%
Malignancy	16	10,80%

**(b):** 

Number of stents implanted	61	
Patients who received a stent (*n*, %)	45	30%
Average stent length	64 mm	
